# Predictors of Improvement in Quality of Life When Treating Hypothyroidism

**DOI:** 10.1155/2021/5577217

**Published:** 2021-06-11

**Authors:** Bjarke Borregaard Medici, Jeppe Lerche la Cour, Filip Krag Knop, Martin Krakauer, Luba Freja Michaelsson, Jens Faber, Torquil Watt, Birte Nygaard

**Affiliations:** ^1^Department of Medicine, Herlev and Gentofte Hospital, University of Copenhagen, Herlev Ringvej 75, Herlev 2730, Denmark; ^2^Center for Clinical Metabolic Research, University of Copenhagen, Gentofte Hospital, Kildegårdsvej 28, Opgang 7, 3. Sal, Hellerup 2900, Denmark; ^3^Department of Clinical Medicine, Faculty of Health and Medical Sciences, University of Copenhagen, Nørre Allé 20, Copenhagen 2200, Denmark; ^4^Novo Nordisk Foundation Center for Basic Metabolic Research, Faculty of Health and Medical Sciences, University of Copenhagen, Copenhagen, Denmark; ^5^Department of Clinical Physiology and Nuclear Medicine, Herlev and Gentofte Hospital, University of Copenhagen, Gentofte Hospitalsvej 2, 1. Sal, th, Hellerup, Denmark

## Abstract

**Background:**

Primary hypothyroidism is characterized by reduced quality of life (QoL). Although thyrotropin (TSH) is utilized as the primary indicator of thyroid disease and treatment adequacy, no simple correlation between QoL and TSH has been shown. This study aimed to investigate changes in clinically relevant predictors during initiation of levothyroxine (L-T4) therapy and their ability to predict improvement in QoL.

**Method:**

Quality of life was measured in patients with newly diagnosed hypothyroidism, during the initial 12 months of L-T4 therapy, by the thyroid-related patient-reported outcome questionnaire, ThyPRO-39. The main outcome measures were the Composite QoL scale and the Tiredness and Emotional Susceptibility subscales (0–100, higher scores worse). Clinical variables (resting energy expenditure (REE), body composition, thyroid function, L-T4 dose, and cognitive function tests) were evaluated as predictors of improvement in QoL by univariate and multiple regression analysis.

**Results:**

Thirty-seven hypothyroid patients with a baseline median TSH of 30 mU/l and a median QoL score of 29 were included. After twelve months of L-T4 treatment, the ThyPRO-39 QoL score had significantly improved to a median score of 14, while REE per kg fat-free mass (FFM) increased significantly from a mean of 26.5 to 28.7 kcal/day/kg (*p* < 0.001). Change in ThyPRO-39 was not associated with a change in REE/FFM (unstandardized coefficient (USC): 0.09 with confidence interval (CI): −1.93 to 2.11, *p*=0.93) but was positively predicted by baseline body mass index (BMI) (USC: 1.54 with CI: 0.59 to 2.49, (*p*=0.002), without association with weight loss (USC: 0.33 with CI: −1.21 to 1.27, *p*=0.96).

**Conclusion:**

Improvement in QoL as measured by ThyPRO-39 after initiation of L-T4 therapy for hypothyroidism was not associated with changes in REE. High baseline BMI, but not weight loss during therapy, was associated with improvement in QoL. This trail is registered with www.Clinicaltrials.gov (registration no. https://clinicaltrials.gov/ct2/show/NCT02891668).

## 1. Introduction

Hypothyroidism is characterized by reduced quality of life (QoL), due to a range of diverse symptoms [1, 2], with tiredness and emotional susceptibility being prominent complaints [3]. Thyrotropin (TSH) is considered the most sensitive marker for evaluating the severity of hypothyroidism. Nevertheless, in the clinical setting, the burden of symptoms does not correlate with TSH concentrations [4–6]. Levothyroxine treatment improves QoL in patients with hypothyroidism, but 5–10% still report symptoms despite adequate levothyroxine (L-T4) dosage [7]. These residual symptoms remain puzzling, though several explanations have been suggested, including insufficient hormone substitution to normalize triiodothyronine (T3) in all tissues, coexistence of other autoimmune conditions, the awareness of being dependent on medicine, and symptoms not originating from thyroid insufficiency [7, 8]. Importantly, overweight is a frequent phenomenon among patients with hypothyroidism and in the general population, and overweight is strongly associated with reduced QoL. The risk of overweight is influenced by many factors, including genetics [9]; nonetheless, daily calorie intake surmounting daily calorie expenditure remains the core problem [10]. Daily calorie expenditure is comprised of dietary-induced thermogenesis (10–15%), physical activity (10–30%), and resting energy expenditure (REE) (60–80%). Therapy with L-T4 significantly increases REE in hypothyroidism [11] due to increased tissue metabolism [12]. Cognitive complaints are common among hypothyroid patients [13], and a connection between cognitive function and QoL may exist, similar to the correlation between cognitive deficits and depression observed in patients with multiple sclerosis [14].

The aim of this study was to investigate changes in clinically relevant predictors (REE, body composition, thyroid function, L-T4 dose, and cognitive function tests) during initiation of L-T4 therapy and their ability to predict improvement in QoL as measured by ThyPRO-39 [15].

## 2. Methods

### 2.1. Study Design

This was a prospective clinical cohort study, including newly diagnosed hypothyroid patients, followed during the initial 12 months of L-T4 therapy. Patients were recruited from a university hospital endocrine outpatient clinic, by advertising and through the Copenhagen General Population Study [16]. Inclusion criteria were diagnosis of primary hypothyroidism with TSH above 10 mU/l measured at two consecutive occasions and age of 20–75 years. Exclusion criteria were hypothyroidism due to thyroid surgery or pituitary disease, other diseases that might interfere with thyroid function, subacute thyroiditis, and pregnancy. The study comprised three identical visits. The first visit was intended to be before initiating L-T4 treatment, albeit 1-2 weeks of low dose L-T4 therapy (≤50 *μ*g per day) was accepted for patients with severe symptoms. Following the first visit, the dose was increased with short intervals towards normalization of TSH. Second and third visits were conducted after 6 and 12 months of L-T4 therapy, respectively.

### 2.2. Outcomes

The primary outcome was a correlation between the increase in REE and QoL measured using the ThyPRO-39 questionnaire. Secondary outcomes encompassed other possible predictors of increase in QoL from therapy: change in body weight, body composition, thyroid parameters, and cognition tests.

### 2.3. Study Procedures

Before each study visit, QoL was measured by participants completing an online version of ThyPRO-39 [15]. ThyPRO-39 scales range 0–100, with higher levels corresponding to more symptoms or more QoL impairment. The Composite score (summarizing all ThyPRO-39 impairment scales), as well as the Tiredness and Emotional Susceptibility scales, was evaluated [17]. Before each visit, participants were fasting from midnight, avoided exercise the preceding night, and arrived in the morning at the hospital by the least physically active way [18]. Body composition (fat mass (FM) and fat-free mass (FFM)) was determined by dual x-ray absorptiometry, each participant using the same scanner for the entire study (Lunar iDXA, GE Healthcare, Fairfield, CT, USA, or Hologic Discovery scanner, Hologic Inc., USA). Bodyweight was measured on a routinely calibrated scale. On each study visit, blood was drawn for analysis of thyroid function. REE was measured by indirect calorimetry for 12 minutes on a CCM Express calorimeter from MGC diagnostics using a facemask after 20 minutes of resting in the supine position [19, 20]. Since REE is particularly dependent on FFM [21, 22], REE was reported and analyzed as REE/FFM (kcal/day/kg). Similarly, the dose of L-T4 is presented as dose/week/FFM (*μ*g/week/kg) [23]. Cognitive function was tested using California Computerized Assessment Package (CalCAP®; Eric Miller, Palm Springs, CA, USA) testing sequential reaction time (SRT) [24], test 1 (SRT1): respond when two numbers in sequence were identical; test 2 (SRT2): respond when two numbers in sequence were in increasing order. Cognitive function was further tested using Symbol Digit Modalities Test scoring 1–100 with a high number indicating better cognitive function [25].

### 2.4. Serum Analyses

It includes thyrotropin (reference range 0.35–4.0 mU/l), thyroxine (T4) (reference range 60–140 nmol/l), free T4 (fT4) (reference range 11.5–22.7 pmol/l), total T3 (reference range 1.0–2.6 nmol/l), and thyroid peroxidase antibodies (anti-TPO) (reference range <60 kU/l). Due to changes in routine T4 assays, seven patients had fT4 measured instead of total T4. All tests were done on ADVIA Centaur XP from Siemens (Ballerup, Denmark) on the day of the study visit.

### 2.5. Calculations and Statistics

Parametric and nonparametric tests were used according to the distribution of data and presented by mean with SD and median with 1^st^ and 3^rd^ quartiles (Q1-Q3), respectively. In linear regression analyses, the ThyPRO-39 Composite Scale was used as the dependent factor to assess the association between predictors and change in QoL. Nonparametric data were analyzed using bootstrapping [26]. Multiple linear regression was used to generate the model with the best ability to predict change in QoL score. Univariate statistics are presented with the unstandardized coefficient (USC) with confidence interval (CI), which is the estimated change in ThyPRO score from visits 1 to 3 when changing one unit of the predicting variable. After excluding variables due to multicollinearity, multiple regression analyses using the forward stepwise method were conducted, including all suspected predictors. Included variables were changes during the study including REE/FFM, log-TSH, and T3; variables from visit 1 were age, body mass index (BMI), sequential reaction times 1 and 2, Symbol Digit Modalities Test, FFM/FM-ratio, and from visit 3, dose of L-T4 per FFM. All statistics were calculated on IBM SPSS statistics data editor version 22.

## 3. Results

**Figure 1:**
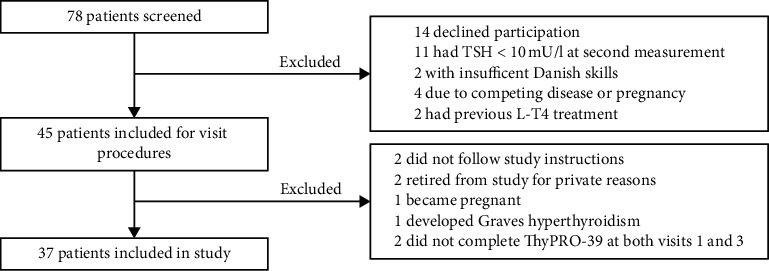
Inclusion flowchart. TSH, thyrotropin; L-T4, levothyroxine.

Seventy-eight hypothyroid patients were screened, and 37 (25 females, mean age 43, SD 13) were included in the study (Figure 1). At visit 2, one patient missed cognitive testing, one patient missed REE-measurement due to technical failure, and four patients did not complete the ThyPRO-39 questionnaire. Data from these patients were only used when comparing visit 1 to visit 3. None of the patients was diagnosed with or treated for depression or other psychiatric diseases during the study.

### 3.1. TSH and Dosage

At the first study visit, median TSH was 33 mU/l (range 10–332), and 36 patients had elevated TPO antibodies. Twenty-seven patients were overtly hypothyroid (elevated TSH combined with T4 and/or fT4 below 70 mmol/l and 12 pmol/l, resp.) with a median TSH level of 75 mU/l, and ten patients were subclinically hypothyroid (elevated TSH with normal T4 and/or fT4 concentrations) with a median TSH of 14 mU/l. Of the 37 patients included, 26 initiated L-T4 therapy after visit 1 (baseline median TSH 28.4 (Q1–Q3: 12.5–111 mU/l)), whereas 11 patients were treated for 1–2 weeks with L-T4 (25–50 *μ*g/day) before visit 1 (baseline median TSH 77 mU/l (Q1–Q3: 16.7–93)). Between visits 1 and 2, TSH, T4, fT4, and T3 levels all normalized and did not significantly change further (Table 1). At visit 2, the mean dosage of L-T4 was 890 (SD 211) *μ*g/week, and at visit 3 the mean dosage was 944 (SD 231) *μ*g/week, with the latter corresponding to 1.7 *μ*g/kg body mass per day or 2.6 *μ*g/kg FFM per day.

### 3.2. REE and QoL

As seen in Table 1, the ThyPRO-39 QoL scale improved by median 14 (Q1–Q3: 3–29) during the study (*p* < 0.001) from visit 1 to visit 3 but did not change significantly between visits 2 and 3 and was not influenced by gender or thyroid status. Both unadjusted REE and REE/FFM increased (*p*=0.002 and *p*=0.001, resp.), with no difference between gender or degree of hypothyroidism (overt/subclinical). REE/FFM was higher among women than men at all visits (*p* < 0.001). Improvement in ThyPRO-39 QoL score did neither correlate (univariate regression analysis) to increase in REE/FFM from visits 1 to 2 nor increase in REE/FFM from visits 1 to 3 (USC 0.09, CI: −1.93 to 2.11, *p*=0.93).

From the forward stepwise multiple regression analysis, we constructed a model including BMI, L-T4 dose at visit 3, and age. The adjusted R-squared value of the model was 0.43 (*p*=0.027). In this model, USC was 2.1 kg/m^2^ for BMI, 15.8 *μ*g/kg FFM/day for dose at visit 3, and −0.5 years for age.

In a multivariate analysis controlling for BMI, age, and L-T4 dose at visit 3, the correlation between the increase in REE/FFM and improvement in ThyPRO-39 remained insignificant (USC −0.77, CI: −2.35 to 0.81, *p*=0.33).

### 3.3. BMI and QoL

Baseline BMI predicted improvement in ThyPRO-39 score from treatment (USC 1.54, CI: 0.59 to 2.49, *p*=0.002) (Table 2) and was not correlated to baseline TSH (*p*=0.998), T4 (*p*=0.39), or T3 (*p*=0.73). At visit 1, BMI correlated with ThyPRO-39 score (USC 1.82, CI: 0.64 to 2.99), but not at visit 2 (USC 0.50, CI: −0.51 to 1.51), nor at visit 3 (USC 0.37, CI: −0.41 to 1.14), while BMI decreased by approximately 0.5 kg/m^2^ from baseline (Table 1). Comparing patients with BMI above or below 25 kg/m^2^, QoL was significantly worse in the high BMI group at baseline (*p*=0.03), whereas QoL was similar between the two groups at visit 3 (*p*=0.36). Thus, the group with high BMI demonstrated a larger improvement in ThyPRO-39 score than the group with normal BMI (25.0 vs. 11.1, *p*=0.04). The two groups did not differ regarding weight loss (*p*=0.3) or REE/FFM during treatment (*p*=0.3). Baseline ThyPRO-39 score significantly predicted improvement in ThyPRO-39 score from visits 1 to 3 (USC 0.7, CI: 0.53 to 0.86, (*p* < 0.001).

### 3.4. Cognitive Function

CalCAP reaction times did not change significantly, but the score from the Symbol Digit Modalities Test did increase from visits 1 to 3 by 4.6 points (*p* < 0.001).

## 4. Discussion

In this study, we looked for clinically relevant predictors of improvement in QoL, as measured by the ThyPRO-39 Composite scale, during initiation of L-T4 treatment in patients with primary hypothyroidism. We hypothesized that changes in REE would be a sensitive predictor for improving QoL during treatment. However, although REE increased, no association with ThyPRO-39 was found. In contrast, BMI at baseline was a strong predictor of improved QoL during treatment, even though patients with high BMI did not lose more weight during treatment compared to patients with BMI below 25 kg/m^2^. Thus, the higher the pretreatment BMI, the better the outcome regarding QoL during L-T4 treatment for hypothyroidism.

Thyrotropin is recognized as the primary measure for evaluating the need for initiating or adjusting L-T4 therapy [26, 27]. However, the ability of TSH to reflect symptoms varies; one study demonstrated a correlation between the number of symptoms and TSH [4], while other studies did not [6, 8]. REE is the product of consumed oxygen and exhaled carbon dioxide [28] and hence directly reflects an integral part of energy metabolism. Considering that REE increases from L-T4 therapy [11], we hypothesized that REE reflects changes in QoL from L-T4 therapy better than TSH, and we, therefore, investigated REE/FFM as a predictor of changes in QoL scores. Our data, which are the first to evaluate this, do not support our hypothesis. Although patients had a significant and clinically relevant increase in QoL score and a significant increase in REE/FFM, the linear regression for REE/FFM as a predictor of QoL improvement was insignificant when controlling for BMI, age, and L-T4 dose/FFM at visit 3. Although this study is of limited size, the range of the confidence interval is widely around zero indicating that the negative result was not due to a type 2 error. No previous study has evaluated a correlation between REE and QoL in hypothyroid patients. As the study has a limited size, we may have overlooked a minor clinical insignificant correlation.

There is a known negative correlation between BMI and QoL in general [29, 30]. Similarly, in the present study, ThyPRO-39 score at baseline was worse among overweight subjects. In our study, we found a correlation between baseline BMI and improvement in ThyPRO-39 during L-T4 treatment, indicating that the higher the BMI is, the more the benefit from L-T4 therapy might be expected. One might argue that baseline ThyPRO-39 should have been included in the multivariate analysis model, and by doing this, baseline ThyPRO-39 independently predicted a reduction in QoL. Further, both age and BMI became insignificant predictors, while L-T4 dose per FFM remained significant (*p*=0.04). However, we chose not to include baseline ThyPRO-39 in the model, since this was not a placebo-controlled randomized trial. Thus, given the design of our study, we cannot estimate the placebo-effect nor the effect from regression towards the mean in QoL, and therefore, we did not include baseline ThyPRO-39 as a predictor of change in ThyPRO-39.

Overweight hypothyroid patients may experience more benefit from L-T4 therapy, due to a lower baseline QoL, which is similar to a meta-analysis demonstrating that patients with more severe depression seem to have greater benefit from both antidepressant and placebo therapy [31].

In other words, patients with the worst symptoms will experience a larger improvement from therapy. This additional benefit may be described as regression towards the mean, comparable to a placebo effect, and we would expect it to dissipate over time.

Studies have demonstrated that the weight loss produced by L-T4 therapy in hypothyroidism primarily originates from loss of FFM [11, 32], and weight gain has even been reported after the initial weight loss [33]. Theoretically, to maintain body weight and composition against an increase in REE of 81 kcal/day (energy intake should increase corresponding to 7.6 kg sugar or 70.4 l regular cola per year), we, therefore, suggest that L-T4 therapy may require a simultaneous diet for a permanent weight loss to occur.

Dosage of L-T4 per FFM was included in the multiple regression model but did not significantly predict change in QoL score in the univariate regression analysis. A higher dosage of L-T4 would, in most cases, lead to a lower TSH in patients. However, TSH and T3 levels were not correlated to change in QoL. These findings are similar to observations from a double-blind crossover trial comparing three different TSH targets, in patients with hypothyroidism. The L-T4 dosage preferred by patients was not determined by TSH, but seemingly by the patients' perception of high L-T4 dose [34].

Cognitive tests did not predict changes in QoL, and only the Symbol Digit Modalities Test score increased significantly between visits 1 and 3. The modest increase in test score was probably due to learning from repeated attempts and not due to increased cognitive performance [25].

A strength of this study is the inclusion of both female and male patients with a TSH > 10 mU/l, all treated according to guidelines. Another strength is the long follow-up that takes into consideration seasonal changes in behavior and mood. Limitations to this study are the limited size, and the open-label design without a control group due to ethical reasons. Further, we did not include T4 or fT4 in regression analyses, as two different assays had been utilized, which is a limitation.

## 5. Conclusion

During the initiation of L-T4 therapy, improvement in QoL as measured on the ThyPRO-39 scale was not associated with changes in REE. In contrast, a higher baseline BMI indicated a better outcome with regard to QoL during L-T4 treatment, whereas the change in BMI during treatment did not seem to affect QoL.

## Figures and Tables

**Table 1:** Changes in thyroid function, QoL, REE, body composition, and cognitive function from L-T4 therapy.

		Visit 1	Visit 2	Visit 3
Thyroid function	TSH (*n* = 37) (mU/l)^*∗*^	33 (14.0; 106)	2.2 (0.9; 4.2) ^1c^	1.5 (0.4; 2.7) ^1c^
	Total T4, *n* = 29 (nmol/l)	59 (29)	122 (19) ^1c^	123 (21) ^1c^
	Free T4, *n* = 7 (pmol/l)	8.0 (2.8)	15.7 (2.0) ^1b^	16.9 (1.9) ^1c^
	Total T3 *n* = 36 (mmol/l)	1.2 (0.48)	1.5 (0.31) ^1b^	1.5 (0.26) ^1c^

QoL (ThyPRO-39) (*n*=37)	Composite score^*∗*^	30 (18; 64)	15 (7; 32) § ^1c^	14 (9; 22) ^1c^
	Tiredness	61 (26.4)	35 (20.5) § ^1c^	35 (19.9) ^1c^
	Emotional susceptibility	40 (29.7)	22 (22.0) § ^1b^	22 (21.5) ^1c^

REE (*n*=35)	REE (kcal/day)	1363 (229)	1445 (245) ^1c^	1446 (229) ^1b^
	REE/FFM (kcal/day/kg)	26.6 (3.6)	28.9 (4.2) ^1c^	28.7 (4.4) ^1c^

Body composition (*n*=36)	Weight (kg)	80.2 (18.7)	79.0 (18.5) ^1a^	79.0 (18.1)
	BMI (kg/m^2^)	27.1 (5.8)	26.6 (5.7) ^1a^	26.6 (5.6)
	Fat mass (kg)	25.6 (10.9)	25.6 (11.1)	25.2 (10.8)
	Fat-free mass (kg)	52.3 (11.5)	51.3 (11.7)	51.8 (11.6)

Cognitive function tests (*n*=35)	SRT1^*∗*^	509 (460; 610)	502 (450; 572)	508 (473; 569)
	SRT2^*∗*^	515 (457; 609)	493 (445; 570)	525 (438; 624)
	SDMT score (*n* = 36)	49.0 (9.1)	52.1 (9.2) ^1c^	53.7 (10.1) ^1c^

Parametric data presented as mean (SD) and nonparametric data if indicated by ^*∗*^ as median (1^st^ and 3^rd^ quartile (Q1–Q3)). ^1a, 1b^ and ^1c^ Significantly different from visit 1 (*p* < 0.05, *p* < 0.01, and *p* < 0.001, resp.); § *n* = 33; TSH, thyrotropin; T4, thyroxine; T3, triiodothyronine; QoL, quality of life; REE, resting energy expenditure; kcal, 1000 calories; BMI, body mass index; FFM, fat-free mass; SRT, sequential reaction time; SDMT, symbol digit modalities test.

**Table 2:** Predictors of improvement in QoL (ThyPRO-39) from visit 1 to visit 3.

	Predictor	Unstandardized coefficient with CI	*p* value
REE	Change in REE/FFM from visits 1 to 3 (kcal/day per kg)	0.09 (−1.93 to 2.11)	0.93

Thyroid function	TSH at visit 1^*∗*^ (mU/l)	0.052 (−0.04 to 0.20)	0.37
	Change in TSH from visits 1 to 3^*∗*^	0.053 (−0.04 to 0.20)	0.33
	Increase in total T3 from visits 1 to 3 (mmol/l)	10.39 (−3.67 to 24.46)	0.14
	T3 at visit 3 (mmol/l)	10.37 (−15.90 to 36.70)	0.43

Dosage	Dosage L-T4 at visit 3 (*μ*g/week)	0.034 (0.01 to 0.06)	0.02
	Dosage L-T4/FFM at visit 3 (*μ*g/kg FFM per week)	1.05 (−0.81 to 2.91)	0.26

Age	Age (yr)	−0.38 (−0.90 to 0.13)	0.14

Body composition	BMI at visit 1(kg/m^2^)	1.54 (0.59 to 2.49)	0.002
	FFM/FM-ratio at visit 1	−6.45 (−12.20 to −0.69)	0.03
	Weight loss between visits 1 and 3 (kg)	0.33 (−1.21 to 1.27)	0.96

Cognitive function tests at visit 1	Sequential reaction time 1^*∗*^ (ms)	−0.03 (−0.10 to 0.04)	0.38
	Sequential reaction time 2^*∗*^ (ms)	0.00 (−0.06 to 0.06)	0.95
	SDMT score at visit 1	0.39 (−0.37 to 1.15)	0.3

^*∗*^ Calculated with bootstrap, CI: confidence interval. The unstandardized coefficient is the estimated change in ThyPRO-39 composite score from visits 1 to 3 when changing one unit of the predicting variable. REE, resting energy expenditure; FFM, fat-free mass; FM, fat mass; kcal, 1000 calories; TSH, thyrotropin; T3, triiodothyronine; L-T4, levothyroxine; BMI, body mass index; SDMT, symbol digit modalities test.

## Data Availability

The data are stored on a secured drive at Herlev Hospital, Denmark. No public data have been utilized.
